# Editorial: Immunological Consequences of Antigen Sampling at Mucosal Surfaces

**DOI:** 10.3389/fimmu.2019.02773

**Published:** 2019-11-26

**Authors:** Neil A. Mabbott, Koji Hase

**Affiliations:** ^1^Royal (Dick) School of Veterinary Studies, The Roslin Institute, University of Edinburgh, Midlothian, United Kingdom; ^2^Division of Biochemistry, Faculty of Pharmacy, Graduate School of Pharmaceutical Science, Keio University, Tokyo, Japan

**Keywords:** mucosal immunity, oral tolerance, M cells, macrophages, dendritic cells, intestinal epithelial cells, mucosal-associated lymphoid tissues, respiratory tract

This Research Topic aimed to provide a useful resource on our current understanding of the mechanisms that mediate antigen sampling at mucosal surfaces, and the influence they have on mucosal immunity. The body's mucosal surfaces such as those lining the gastrointestinal and respiratory tracts are continuously exposed to large burdens of commensal bacteria, as wells as pathogenic microorganisms and their toxins (1). As a consequence, the mucosal immune system has evolved to enable the body to induce specific immunity toward pathogenic microorganisms, and also mediate tolerance toward commensal bacteria and food-derived antigens (2). For example, production of antigen-specific secretory IgA in mammalian gut-associated lymphoid tissues (GALT) such as the Peyer' patches provides protection against certain pathogens, as well as maintaining homeostasis in the intestinal commensal microbiota (3). To achieve this, unique mononuclear phagocyte populations in the lamina propria and specialized epithelial cells actively sample the lumenal contents to deliver antigens into the mucosal-associated lymphoid tissues to induce tolerance or trigger antigen-specific immune responses.

Much is known of how the commensal microbiota can affect IgA production in the intestine (4, 5), but in this collection Hara et al. describe the influence that dietary antigens may also have. They show that mice fed antigen free diets had reduced intestinal IgA production, and reduced numbers of germinal center B cells and T follicular helper (Tfh) cells in their Peyer's patches. Importantly, dietary antigens also appear to play a role in host protection against intestinal pathogens, affecting for example, immunity to *Salmonella* Typhimurium infection.

In their study, Zhao et al. define the mechanism by which intranasal chitosan-formulated DNA vaccination induces IgA secretion in the intestine. They show that chitosan-DNA is specifically targeted to CD103+ dendritic cells. These cells accumulate in the mesenteric lymph nodes and promote IgA-class switching in antigen-specific B cells via IL-6-IL-6R and BAFF-TACI signaling, as well as Th17/Tfh cell differentiation. These data suggest that intranasal chitosan delivery may have useful adjuvant application for the induction of intestinal IgA responses.

The expression of major histocompatibility complex II (MHC-II) by intestinal epithelial cells (IEC) raises the suggestion that IEC contribute to immunity or tolerance through MHC-II-mediated antigen presentation to T cells (6). However, testing this hypothesis in the human intestine is hindered by a lack of physiologically-relevant *in vitro* systems. Wosen et al. show how IFNγ stimulation can induce MHC-II expression in human small intestinal enteroids (“mini guts”). Since enteroids could be derived from individual patients with distinct MHC-II genotypes this may represent a novel tractable *in vitro* system to interrogate the role of IEC-expressed MHC-II in celiac disease and other intestinal MHC-II-associated diseases.

In the intestine specialized antigen-sampling IEC known as M cells (or microfold cells) reside within the follicle-associated epithelia (FAE) covering the GALT. Due to many important recent advances it is perhaps not too surprising that articles covering M-cell immunobiology feature strongly in this Research Topic. Dillon and Lo provide a detailed review of the cellular and molecular factors that regulate M-cell differentiation in the mucosal-associated lymphoid tissues. M cells constitutively sample the lumenal contents and rapidly transcytose particulate antigens across their short cytoplasm into the underlying basolateral pocket where they are sampled by leukocytes and lymphocytes. This review also contains an in-depth description of the unique molecular and morphological features that enable M cells achieve their important immune surveillance functions.

Much of our knowledge of M cells was derived from the analysis of cells and tissues during the steady-state. Dillon and Lo also provide a comparative account of how M cells can be induced under certain circumstances in the chronically inflamed intestine. Complex interactions between host genetic and environmental factors can profoundly affect the pathogenesis of some autoimmune diseases. Since dysbiosis of the intestinal microbiota is increasingly identified as one of these factors, Kobayashi et al. discuss the novel hypothesis that dysbiosis and M-cell mediated bacterial transport into Peyer's patches may also affect susceptibility to, or the pathology of, some autoimmune diseases. For example, they propose how the transcytosis of segmented filamentous bacteria by M cells might induce Tfh cells differentiation in Peyer's patches, and how this might stimulate autoantibody production, and by doing so exacerbate the pathogenesis of arthritis. The exciting array of M cell-specific tools that are now available provide timely opportunities to test the many interesting hypotheses raised in this review.

Cells with M cell-like characteristics have also been described in the FAE lining the nasopharynx-associated lymphoid tissues of the upper respiratory tract (7). In their study, Kimura et al. describe the identification and features of M cells associated with the tracheal and bronchial epithelia of the lower respiratory tract. Interestingly, M cells were also observed in the epithelia associated with ectopically-induced lymphoid follicles in distinct mouse models of respiratory disease. Further studies are now required to determine the role these cells play in the induction of immunity in the respiratory tract and their influence in respiratory disease pathogenesis.

Colony-stimulating factor 1 (CSF1) controls the growth and differentiation of macrophages (8). Furthermore, expression of the CSF1 receptor (CSF1R) protein in mammals is restricted to the mononuclear phagocyte lineage and is not expressed in any IEC lineage, including M cells in the gastrointestinal tract (9). Balic et al. report the surprising finding that M cell-like cells in the FAE covering the bursa of Fabricius of avian species such as the chicken express CSF1R highly. This raises the hypothesis that in contrast to mammals the differentiation avian M cell-like cells may directly depend on CSF1-CSF1R stimulation. The demonstration that avian M cell-like cells express CSF1R highly also provides a useful novel marker to specifically study their role in mucosal immunity and disease.

In conclusion, we consider the novel advances and stimulating hypotheses presented within these articles will provide useful foundations for future studies on the factors that mediate antigen sampling at mucosal surfaces to improve tolerance, prevent disease and enhance mucosal immunity to pathogen infections.

## Author Contributions

NM and KH wrote the manuscript, edited the manuscript, and approved the final submitted version of the text.

### Conflict of Interest

The authors declare that the research was conducted in the absence of any commercial or financial relationships that could be construed as a potential conflict of interest.

## References

[1] Sender R Fuchs S Milo R. Are we really vastly outnumbered? Revisiting the ratio of bacterial to host cells in humans. Cell. (2016) 164:337–40. 10.1016/j.cell.2016.01.013 26824647

[2] Mowat AM Anatomical basis of tolerance and immunity to intestinal pathogens. Nat Rev Immunol. (2003) 3:331–41. 10.1038/nri1057 12669023

[3] Rios D Wood MB Li J Chassaing B Gewirtz AT Williams IR. Antigen sampling by intestinal M cells is the principal pathway initiating mucosal IgA production to commensal enteric bacteria. Mucosal Immunol. (2016) 9:907–16. 10.1038/mi.2015.121 26601902 PMC4917673

[4] Furusawa Y Obata Y Fukuda S Endo TA Nakato G Takahashi D. Commensal microbe-derived butyrate induces the differentiation of colonic regulatory T cells. Nature. (2013) 504:446–50. 10.1038/nature12721 24226770

[5] Ivanov II Honda K. Intestinal commensal microbes as immune modulators. Cell Host Microbe. (2012) 12:496–508. 10.1016/j.chom.2012.09.009 23084918 PMC3516493

[6] Wosen JE Mukhopadhyay D Macaubas C Mellins ED. Epithelial MHC class II expression and its role in antigen presentation in the gastrointestinal and respiratory tracts. Front Immunol. (2019) 9:2144. 10.3389/fimmu.2018.02144 30319613 PMC6167424

[7] Date Y Ebisawa M Fukuda S Shima H Obata F Takahashi D. NALT M cells are important for immune induction for the common mucosal immune system. Int Immunol. (2017) 29:471–8. 10.1093/intimm/dxx064 29186424

[8] Dai XM Ryan GR Hapel AJ Domingues MG Russell RG Kapp S. Targeted disruption of the mouse colony-stimulating factor 1 receptor gene results in osteopetrosis, mononuclear phagocyte deficiency, increased primitive progenitor cell frequencies, and reproductive defects. Blood. (2002) 99:111–20. 10.1182/blood.V99.1.111 11756160

[9] Sehgal A Donaldson DS Pridans C Sauter KA Hume DA Mabbott NA. The role of CSF1R-dependent macriophages in control of the intestinal stem-cell niche. Nat Commun. (2018) 9:1272. 10.1038/s41467-018-03638-6 29593242 PMC5871851

